# Changing homelessness services: revanchism, ‘professionalisation’ and resistance

**DOI:** 10.1111/hsc.12159

**Published:** 2014-12-01

**Authors:** Lisa Scullion, Peter Somerville, Philip Brown, Gareth Morris

**Affiliations:** 1 School of Nursing, Midwifery Social Work & Social Sciences, University of SalfordSalford, UK; 2 School of Social and Political Sciences, University of LincolnLincoln, UK

**Keywords:** collaboration, communities of practice, homelessness, leadership, ‘professionalisation’

## Abstract

This paper argues that the increasing international salience of homelessness can be partially explained by reference to the revanchist thesis (involving processes of coerced exclusion and abjection), but the situation on the ground is more complex. It reports on interviews with 18 representatives of 11 homelessness service providers in one city in England. As Cloke *et al*. found, these providers tended to be either larger, more ‘professional’, ‘insider’ services or smaller, more ‘amateur’, ‘outsider’ services. However, this does not mean that the former were necessarily more revanchist and the latter less so. Rather, the actions of both types of organisation could, in some cases, be construed as both advancing and counteracting a revanchist project.

## What is known about this topic

- Homelessness is complex and multidimensional.
- Service provision for homeless people is extremely uneven, nationally and internationally, in terms of location, type and quality of service.
- Collaboration among different agencies is typically very difficult, but not impossible, to achieve.

## What this paper adds

- The revanchist thesis is re-evaluated in relation to homelessness.
- Effective service for homeless people requires leadership both from larger organisations at a strategic level and from keyworkers at an operational level (co-ordinating different services and advocating for their clients), which can be successfully integrated within self-organising communities of practice.
- Self-organising services are continually threatened by sometimes well-intentioned, but often misguided, government intervention.

## Introduction

Homelessness emerged as a major global issue in the 1980s (Davis 1990, Takahashi 1996, Mitchell 1997, Wacquant 1999), coinciding with the ascendancy of neoliberalism, associated in the United States with cuts in federal welfare, and in the United Kingdom, with mass unemployment particularly for young people (Furlong & Cartmel 2007). The authorities' response to homelessness, most notably in New York (Zukin 1995, Duneier 1999), was antagonistic, oppressive and punitive – part of an allegedly revanchist project (an attempt by more powerful groups in society to regain, by force if necessary, their territorial domination over less powerful groups) to reclaim prime spaces (such as parks, train stations and pavements) from homeless people, for the benefit of international capital (Smith 1996, Mitchell 1997, 2003, pp. 163–167), local businesses and affluent consumers, or to deliver ‘clean streets’ (Mitchell 2003, Cloke *et al*. 2010, p. 242). Homeless people excluded by this response were to be incarcerated (or warehoused), spatially contained or entirely displaced from the area (Mitchell 2001).

Some scholars have questioned the revanchist thesis. DeVerteuil (2006, p. 111) pointed out that the ‘archetypal revanchist city’, New York, provided substantial funding for shelters that support rather than oppress or punish homeless people. Research does not detect a ‘co-ordinated punitive regime’ (DeVerteuil 2006, p. 118), but rather a relatively uncoordinated combination of supportive and punitive measures, a new ‘poverty management’ (DeVerteuil 2006, pp. 118–119) involving the ‘re-institutionalisation and circulation’ of homeless people as well as their removal from prime urban locations. The key institutions are shelters, which are ‘contradictory and nuanced institutions that contain/conceal/manage the homeless while also providing basic subsistence needs and hopefully some prevention’ (DeVerteuil 2006, p. 119).

On closer inspection, however, the new ‘poverty management’ seems at least compatible with revanchism, which typically involves *coercion* of homeless people, in their exclusion from prime spaces and their abjection (cast down as well as out) (Kristeva 1982). This abjection, however, is not necessarily permanent or intended to be so; it can be associated with containment, confinement or ‘maintenance’ (in abeyance) within marginal spaces such as shelters or ghettoes (Wacquant 2008), where the abject are subjected to a variety of disciplines, crudely understood as ‘care and control’. This is evident in the imposition of market disciplines (Cloke *et al*. 2010, p. 37) and control of the ‘conduct of conduct’ (Cloke *et al*. 2010, p. 38). This ‘coercive care’ (Johnsen & Fitzpatrick 2010) is justified in terms of a ‘civilising’ (Uitermark & Duyvendak 2008, Flint & Powell 2009), ‘modernising’ (Newman 2001) or ‘securitarian’ (Dikeç 2013) offensive. The aim was to eradicate the ‘sin’, not the ‘sinner’, who is to be cleansed and ‘transfigured’ (Cloke *et al*. 2010, p. 43). Thus, despite continuing shortages both of relevant facilities and of move-on accommodation (Cloke *et al*. 2010, p. 38), homeless people can still be rehabilitated into mainstream society, where they have their own permanent, self-contained accommodation (DeVerteuil *et al*. 2009, p. 653).

The significance of the revanchist thesis, therefore, lies primarily in its emphasis on processes of coerced exclusion and abjection, and secondarily on the management of the consequences of these processes. The evidence, however, is mixed. In some US cities, it seems that homeless people have little option other than to enter shelters and other forms of institution where they are subjected to ‘a hybrid of sin-talk and sick-talk – moralism and pop-medicalization’ (Gowan 2010, p. 287), to convert them into active, responsible citizens. In general, however, services are ‘fragmented’ and ‘diffuse’ (DeVerteuil 2006, p. 113); highly uneven in terms of form, content, quality and distribution (DeVerteuil 2006, p. 117, DeVerteuil *et al*. 2009, p. 654, Cloke *et al*. 2010, p. 9); and both low-support and high-support services vary in the quality of care they provide (DeVerteuil *et al*. 2009, p. 653, Cloke *et al*. 2010).

Cloke *et al*. (2010, p. 57, 59, 252) in particular point to the existence of smaller, ‘outsider’ organisations (Cloke *et al*. 2010, p. 39) that provide ‘service without strings’ (Cloke *et al*. 2010, p. 42) – a ‘voluntary attitude’ (Cloke *et al*. 2010, p. 245) that involves acceptance of and being *for* the other (Cloke *et al*. 2010, p. 60), with no expectation of reciprocity (Cloke *et al*. 2010, p. 58), ‘spontaneous acts of kindness’ (Cloke *et al*. 2010, p. 245) that enable dialogue and the building of relationship. These organisations are likely to provide appropriate and enduring assistance to their clients, incompatible with revanchism. In contrast, larger, ‘insider’ services exist that lack this ‘receptive generosity’ (Cloke *et al*. 2010, p. 52), and expect homeless people to make specific changes in how they behave and live. Also, as these organisations become more governmentalised (funded by government contracts and subjected to government policy and regulation), they tend to become more ‘professionalised’, meaning they become more skilled at meeting the needs prioritised by government (Cloke *et al*. 2010, p. 45, and see discussion below on Stoke). Such professionalisation can undermine their voluntary attitude (Cloke *et al*. 2010, p. 250), in particular their role as advocates of homeless people (Cloke *et al*. 2010, p. 244, 251). This does not amount to revanchism; however, ‘professionals’ can have a voluntary attitude, while ‘amateurs’ can lack receptivity to the other. The actions of any given professional or amateur can *simultaneously* both advance (through containment and discipline) and push back (through meeting physical, emotional and spiritual needs) a revanchist project. In general, as organisations within capitalism, the homelessness industry works to prepare its clients for employment, in which they produce commodities and services that have value both in supporting and expanding the power of capital and in meeting needs and possibly challenging that power.

Attention has focused on ‘archipelagos’ of agencies (Gowan 2010) in different countries working with homeless people in diverse fields including health, criminal justice, social work, education and employment, as well as housing (for the UK, see Glover-Thomas 2007). Despite the use of one-stop shops (Pannell & Parry 1999, on *The Hub* in Bristol; DeVerteuil 2006, pp. 114–115, on ‘mega-shelters’ in Los Angeles County), both service users *and* providers face difficulties in navigating through the various services (Roche 2004). Regardless of the political project affecting homelessness (revanchism, neoliberalism, welfarism or postsecularism), substantial evidence indicates serious practical obstacles to achieving service co-ordination, including conflicting organisational priorities (reflecting different government targets and the requirements of different funding bodies), a competitive funding system, and general scarcity of resources and trust (Milbourne 2009, p. 281). Even within the same organisation, priorities may conflict, for example, between housing regeneration and the provision of affordable housing and social services for low-income households (DeVerteuil *et al*. 2009, p. 651, Williams & Sullivan 2010).

Even when services are successfully co-ordinated, people more entrenched in homelessness become ‘routinely excluded from effective contact with services they need’ (MEAM, 2009, p. 9) – because their needs are not deemed to require support or are not formally diagnosed, or the services cannot cope with them or take responsibility for them. In the United Kingdom, four major national charities working with homeless people (Homeless Link, Clinks, DrugScope and Mind) formed the Making Every Adult Matter (MEAM) coalition to address this failure to meet the needs of an estimated 60,000 people in the United Kingdom whom it described as having ‘multiple problems’, ‘ineffective contact’ with services and ‘chaotic lives’ (Page & Hilbery 2011, p. 13). This coalition recommended that a named individual should be responsible in every local area for ensuring the support required and that the public be given a ‘right of inquiry’ to ask that individual to provide information about the level of support given to someone with multiple needs. The value of such co-ordination, advocacy and empowerment is clear from other studies (e.g. Milbourne 2009, p. 285, Cattell *et al*. 2011, p. 1).

Such co-ordination and empowerment, however, are swimming against the tide of policy approaches in the United Kingdom and other countries, which give high priority to market forces and low priority to the needs of people who lack market power or political clout and are seen as more ‘difficult’ and, in many cases, less ‘deserving’ of public support. The UK government's main response (HM Government 2012) recognised (for the first time) a subset of people with multiple needs, a need for improved local service co-ordination and ‘the provision of key workers to provide long-term tailored support’ (HM Government 2012, p. 11). This report, however, treated each need separately (crime, substance misuse, poor health, debt, etc.), and passed the responsibility for delivering the desired co-ordination and user empowerment entirely to ‘local leaders’ (HM Government 2012, p. 61). For those deemed capable of it, work was prescribed as the only means of empowerment, while for those unable to work, the report offered only the prospect of undefined ‘recovery’ and ‘reintegration into family and community’ (HM Government 2012, p. 47) – even though ‘recovery’ in the short term can be at the expense of maintenance and improved outcomes in the longer term (Cornes *et al*. 2013, p. 9).

Local leadership, therefore, emerges as key to both co-ordinating services and empowering users. But what is this? Williams and Sullivan (2010, pp. 9–10) propose ‘collaborative leadership’, which involves a variety of roles or activities: building partnerships, recognising the interconnections behind those partnerships, appreciating the contributions made by different partners, and being open to new ideas and practices. It is not clear, however, who is best equipped to exercise this leadership, why co-ordination requires interaction (involving interpersonal relationships) or indeed how this leadership leads to user empowerment. Williams and Sullivan envisage that the leadership role is taken by keyworkers, who embody the meaning and purpose of the collaborative project and work as ‘boundary spanners’ (Williams & Sullivan 2010, p. 11), advocating on behalf of their clients, persuading the different agencies involved and co-ordinating the activities of those agencies. This, however, seems to conflate a variety of roles: providing overall direction, directly influencing, co-ordinating and advocating. Of these roles, only advocacy clearly involves receptivity to the other, whereas the others all look like functions of ‘poverty management’ (understood as the management of the consequences of coerced exclusion and abjection), as discussed earlier. Where collaborative leadership was found to occur, the learning tended to be ‘concentrated in a relatively small cadre of individuals who happened to be associated with a particular collaborative project’ (Williams & Sullivan 2010, p. 12), and risked being lost when those individuals moved on (see also Milbourne 2009, on the problems of collaborating in a competitive environment). It seems that no easy technical or managerial solution exists to the problems of service co-ordination and user empowerment. The only realistic alternative is a *political* solution, in which keyworker advocacy is supported by stable institutional arrangements.

Communities of practice, defined as ‘groups of people who share a concern or passion for something they do and learn how to do it better as they interact regularly’ (Wenger 2006), have been suggested as valuable for improving inter-agency working (Cornes *et al*. 2013). Three elements are said to be required for such a community: an identity based on commitment to developing individual competence in a shared domain of interest; interaction to build relationships that enable mutual learning; and concern for developing collective competence in the sharing of practice as well as interests. It is not clear, however, how much services need to interact with and understand one another rather than just act in concert (i.e. co-ordinated). It might be sufficient and more practicable to rely on keyworkers spanning the boundaries of the agencies and ensuring that the right kind of help (from whatever agency) is provided at the right time for their clients, with strategic groups (such as in Stoke – see below) ensuring that continuity is maintained despite changes of staff, government policy and individual agencies – in short, a keyworker model of advocacy at an operational level, in combination with a politically driven multi-agency grouping at a strategic level.

## Methods and background

This paper presents findings from 18 interviews conducted during spring/summer 2010, and four follow-up interviews in summer 2013, with representatives from key organisations working with homeless and multiply excluded individuals in Stoke-on-Trent. These interviews represented the first stage in a wider research project, and aimed to understand the context of homelessness in the city. The second stage aimed to identify key themes in the life histories of people experiencing multiple exclusion homelessness, including episodes of homelessness across the life-cycle, and involved interviewing 104 homeless and multiply excluded individuals. This paper draws exclusively on the first-stage interviews with key service providers. Analysis of the life histories of people experiencing homelessness is ongoing and will feature in separate publications.

Some organisations had a variety of specialisms (e.g. some provided services for all or a combination of the following: young people, older people, drug and alcohol users, lone parents, refugees and sex workers); so in these cases, it was necessary to interview more than one representative to be able to reflect the diversity of roles and client groups. Consequently, the key informants represented 2 statutory organisations (5 interviewees) and 9 third sector organisations (17 interviewees). The third sector organisations comprised so-called ‘big players’ (9 interviewees) and ‘smaller players’ (8 interviewees) (see below). Overall, the organisations formed a ‘continuum of care’ (Berman & West 1997) in homelessness services, including prevention, emergency accommodation, training and housing support – thus, including the type of service co-ordination, based on receptivity to the other, advocated by Cloke *et al*. (2010, p. 144).

All interviews were audio-recorded – with respondents’ permission – transcribed verbatim and entered into NVivo. Thematic analysis was undertaken on the transcripts, with particular focus on the following: interviewees’ understanding of the causes of homelessness; the particular specialism(s) of their organisation, if relevant; experiences of multi-agency working; perceived good practice in relation to services; and perceived gaps in provision. While our initial approach was based on searching for themes that would help us – in the context of the wider research project – to understand homelessness and homelessness provision across the city, our analysis uncovered a range of other issues concerning the relationships among service providers. It is these issues that form the focus of this paper.

The paper also draws on discussions at the research project advisory group meetings, which were attended by key stakeholders and service providers. While the main purpose of these meetings was for the authors to seek guidance on the project and feed back emerging findings, it was also a useful forum for discussing how services have responded to homelessness in the city, as well as observing the interaction among service providers around particular issues.

## Partnership working in Stoke-on-Trent

This section explores respondents' perceptions of how agencies came to work more closely together, considers how the ‘professionalisation’ of services has occurred, and how larger and smaller organisations are responding to the needs of homeless individuals within the city.

### The trigger for co-operation and the development of a Priority Needs Group

Many factors shape the response to homelessness locally. Cloke *et al*. (2010, p. 12), for example, identify geographical and political factors, while Berman and West (1997, p. 313) refer to particular events and community circumstances as ‘driving forces’ in developing homelessness initiatives. A key such event in Stoke was the death of a homeless man and woman in a fire in a derelict building in 2007. Similar events had occurred before, but in this case, two children were convicted of arson and the homeless people were vividly portrayed as victims by the local media, particularly the *Evening Sentinel*. These circumstances transformed an everyday tragedy into a politically salient event that stimulated decisive action (as Moseley 2009, concluded, inter-agency co-ordination can only be achieved by *political* means, not by technical or managerial reforms).

Our research found that this tragedy was the catalyst for the development of a *Priority Needs Group*, which focused initially on making empty buildings safe and secure to prevent further deaths. A central telephone number was set up and publicised for rough sleepers to contact, together with a protocol of mutual reporting among the Group members concerning homeless people at risk. The Rough Sleepers Team undertook to visit within 24 hours of receipt of any such report. Key members of the Group included Brighter Futures (responsible for the Rough Sleepers Team), the Fire Service, and the Council's Housing and Social Services Departments. This soon extended to include nearly all supported housing providers in Stoke, the police and the probation service. The Group developed common practices, for example, on performance standards and staff training and development. Overall, this response can be represented as a rejection of what might be called the revanchist attitude (which would have regarded the homeless couple as simply unlucky) and a positive embracing of the other by the political community of the city.

The Group continues to hold meetings regularly throughout the year, fortnightly during the winter months, at which it reviews in detail all the most urgent homelessness cases (the nature of the need, the client's history, the involvement of different services, the options available for that client, etc.) and decides what action to take. This illustrates how agencies can come together with a common purpose by focusing greater attention on the needs of people sleeping rough, thus achieving the targeted and co-ordinated services envisaged by MEAM, with Brighter Futures, by virtue of its responsibility for the Rough Sleepers Team, acting as the co-ordinator (Page & Hilbery 2011, p. 3). Although the trigger for action was political, the motivation for forming and maintaining the Group appeared to come from within the agencies themselves. This is why the ‘homelessness industry’ (Ravenhill 2008) in Stoke seems more cohesive than in other cities (for example, Southampton – Buckingham 2009, p. 247). This spontaneously developed community of practice, involving active collaboration, not just co-ordination, was appreciated by both statutory and voluntary organisation participants in the research.

Group members added that since its inception it had ‘branched out’ to focus on specific categories of client such as women (Voluntary organisation representative 5); and sex workers, people with drug and/or alcohol dependencies, and people with a history of violent behaviour (all identified by Reeve *et al*. 2009). It was clear that this community of practice had helped organisations to adapt their services to recognised needs (Edgar *et al*. 1999), thus providing appropriate responses for different homeless populations.

### ‘Professionalisation’ of homelessness provision: a consortium of ‘big players’

The process of ‘professionalisation’ mentioned earlier has mostly involved bureaucratisation and managerialisation, in which relatively informal voluntary arrangements for helping those in need have become replaced by more formal line management control structures, with standardised procedures, paid staff and associated systems of monitoring and administration (Buckingham 2009, p. 245). In the United Kingdom, this process was shaped significantly by the introduction of the Supporting People programme in 2003, which favoured ‘larger, professionalised voluntary organisations and social enterprises’ (Buckingham 2009, p. 244). Although standardisation improved the efficiency and effectiveness of services in some respects (Buckingham 2009, p. 248), it also led to the marginalisation or ‘squeezing out’ (Buckingham 2010, p. 14) of services that provided more for what could be called ‘non-standard’ clients (see below).

This process was reinforced by continuing competition for statutory funding and emphasis on performance management (Fear & Barnett 2003, Barnett & Barnett 2006, Cloutier-Fisher & Skinner 2006, Cloke *et al*. 2010, p. 181), which favoured ‘bigger, better-resourced organisations, while concealing the advantages that small community organisations offer’ (Milbourne 2009, p. 290). In Stoke, core strategy groups for single homeless people were set up, relating to social exclusion, care and support, and care and independence, all with voluntary organisation representation. Around the same time, a consortium of the larger voluntary organisations developed from a re-commissioning of the statutory floating support services (services provided to clients in their own homes, but from outside those homes):

…the thinking was that if we commissioned a service that has got three partners we get a broader range of service delivery rather than one organisation doing floating support and another organisation doing floating support, more often than not they were working with the same people, but they weren't talking to each other. (Voluntary organisation representative 6)

The consortium catered for a diversity of needs and experiences, but was also important for resource acquisition, for example, through successful bidding for contracts (Edgar *et al*. 1999). Its members soon became known as the ‘big players’ in Stoke's homelessness industry, and came to share physical space as well as best practice. They had managers working out of one main office, but also their own staff based in branch offices across the city. This facilitated sharing of knowledge, joint visits and team working generally.

The consortium appears to be a self-selected group based on its members' greater size, power and influence. It effectively formed an ‘inner circle’ within the Priority Needs Group, providing continuity and stability to that industry by achieving co-ordination and collaboration of all relevant service providers. Although not a community of practice in itself, it looks like *an elite group operating within the community of practice* that is the Priority Needs Group. This arrangement seems to ensure that collaborative learning is retained (Williams & Sullivan 2010, p. 12).

The ‘professionalisation’ of homelessness services has involved demonstrating reductions in rough sleeping and movement of clients into accommodation (Cloke *et al*. 2010, p. 33). Inevitably, this creates a tendency to ‘select those who are more likely to contribute to achieving the targets and to ignore the most difficult cases’ (Edgar *et al*. 1999, p. 67, see also MEAM 2009, Milbourne 2009, p. 288, Buckingham 2010, p. 14). Consequently, the consortium members had seen changes in their client profile. As one respondent expressed it:

…the people that we used to accept were more chaotic [and therefore more likely to fail], whereas now, we've got certain expectations, and there's contractual targets that our funders expect us to meet. So if we feel that somebody is too chaotic … we would signpost them to other organisations and we're very rigorous with the supporting information that we gather about a person before we accept them … the clients have to be more stable and more willing to engage with support whereas a few years ago, we would accept anybody really. (Voluntary organisation representative 1)

This quote sits uncomfortably with the Priority Needs Group's aim to address the most complex and difficult cases and suggests that one of the ‘big three’ has gone its own way. It reveals the tension between focusing on the most entrenched (or chaotic) cases and giving preference to clients with fewer or less intractable problems. The shift from the former to the latter is being driven partly by the increasing need to demonstrate cost-effectiveness to funders, particularly governments, and HM Government (2012) is just one more example of this, with its emphasis on ‘recovery’ and on getting clients into work.

The stress on localism (local leadership, local discretion, etc.), however, means that homelessness organisations can resist these pressures to some extent. Conversations with representatives of statutory organisations, particularly the City Council, suggest that they do not recognise the existence of this tension within and among the non-statutory organisations. City Council spokespersons declared that they made referrals of homeless clients to temporary accommodation based entirely on the clients' needs and their knowledge of the services available, though they were of course constrained by whatever vacancies happened to exist at the time. They recognised the protocol for Supporting People providers, but stated that it did not affect their referral decisions. One representative suggested that criteria for entry to some hostels could be difficult, e.g. because they stipulated a single point of access that was not always there, but this seemed to be related to the general practicalities of accessing a scarce resource rather than to any substantive differences in approach within the non-statutory sector (e.g. between the ‘big’ and ‘small’ players). Given this, it may be that the comments from the voluntary organisation representative in the above quote relate to clients who self-refer rather than to those who are referred to that organisation by the City Council.

In support of this, it was clear that the big players, and perhaps some of the smaller players too, mainly operated a keyworker model, in the sense envisaged by Page and Hilbery (2011) and Cattell *et al*. (2011), i.e. a worker who advocates between service users and local services to ensure that they access the right services at the right times. Many referrals to homelessness agencies in Stoke came via the Rough Sleepers Team, who used their own intelligence and information from other service users to identify those in need, although other cases were referred by housing, social services, and the police and fire services. Each case was then normally assigned a keyworker at their point of contact with the accepting organisation.

To conclude, it seems that in Stoke, the core strategy groups, the Priority Needs Group and the consortium of leading players together constitute a community of practice that achieves co-ordination of services to homeless people at both strategic and operational levels, with an emerging emphasis on user empowerment. They are heavily implicated in government systems, programmes and projects, but they are not inherently revanchist because at least some of them are clearly receptive to the other. They are involved in both containment and rehabilitation, but they see the containment as a necessary discipline in order for users to be empowered. In a sense, they are both oppressive and caring – they are both complicit in and resistant to neoliberal projects, including revanchism. Their oppressing, however, has a higher purpose. Like all of us, they are living in contradiction under capitalism, but the balance of their action lies towards the side of the angels. This situation is inevitably very delicate and fragile, and the path towards rehabilitation is fraught with difficulties and setbacks. Receptivity to the other can never guarantee the success of this process, but may be a necessary condition for it.

### The position of the ‘smaller players’

‘Smaller players’ work with very small budgets, often reliant on volunteers (see also Milbourne 2009, p. 288), and are consequently sometimes dismissed as ‘amateur players’ (Cloke *et al*. 2010, p. 46). They are seen as lacking both time and skills to compete in bidding (Milbourne 2009, p. 287), unable to influence the rules of engagement (Milbourne 2009, p. 290), and are generally under-valued by more powerful organisations, including government.

The distinction between bigger and smaller players seems to support the thesis of a bifurcation within the third sector between ‘professionalised, corporatist organisations’ and ‘grassroots, voluntaristic organisations’ (Buckingham 2010, p. 14, see also Cloke *et al*. 2010, p. 40). Buckingham's model of third sector organisations seems to suggest that smaller players are less professionalised and more voluntaristic, and therefore more likely to display a ‘voluntary attitude’ (as discussed earlier) and to be receptive to the other. In Stoke, however, the situation was more complicated than this: first, the big players themselves demonstrated receptivity to the other; and second, interviews with representatives of the smaller organisations suggested that some of them were becoming more ‘professional’, though in a different sense:

There's a lot of tenders going out to these massive companies where it's sort of effectively one service provider for one type of service… When guys present to us they've nine times out of ten burnt their bridges with the [larger organisations] and they're coming to us and they're saying, ‘I know I've cocked up in the past, I know this is my last chance. If I don't do it here I'm on the streets' … You can't just have one service, one super company, providing all the homeless bed spaces in the city because that excludes people once they've made a few mistakes, which they do because they're a chaotic and troubled client group. (Voluntary organisation representative 7)

This quote suggests that some smaller organisations have not bureaucratised or managerialised or corporatised, but have become more focused on addressing complex needs and providing a more personalised service. These organisations provide an ‘environment of choice’ for homeless people (Cloke *et al*. 2010), offering an alternative or, in some cases, a second (or last) chance for certain client groups. The quote also seems to imply that the big players are not being sufficiently receptive to the other (see the earlier quote from voluntary organisation representative 1), and that this lack of receptivity is related to the monopoly that these players currently enjoy over homelessness services provision (‘one service, one super company’).

Thus, while the consortium represented the ‘inner circle’ of the Priority Needs Group, the smaller homelessness organisations formed part of the ‘outer circle’ – not outside the community altogether because they are, increasingly, included within policy and practice, but nevertheless on the *periphery* of what now counts as mainstream provision. The formation of the consortium, therefore, appears to have been associated with a division of labour among third sector organisations (as in Southampton – Buckingham 2010, p. 13) within the Priority Needs Group, which has both advantages and disadvantages. The benefits include a more comprehensive, consistent, co-ordinated and effective service for homeless people with multiple and complex needs, while the drawbacks are that the responsibility for meeting those needs has been partly devolved to less powerful organisations (Buckingham 2010, p. 14). The latter appear to have more influence from within the system now than before, when they were (effectively) excluded from it, but inevitably that influence is less than that exerted by the ‘big players’. The situation is unlikely to change significantly because the key funders, such as governments, seem to be unaware of the potential problem of institutionalising inequality of treatment for people with multiple and complex needs (for further reflection on the role of these smaller players, see Buckingham 2010, p. 16). This increases the risk of further marginalisation of the latter population, as envisaged by the revanchist thesis, and exemplified in the UK government's No Second Night Out initiative, which gives priority to newly homeless people (HM Government 2011).

Many ‘smaller players’, in Stoke as elsewhere, are faith-based and highly committed (with an ethos of ‘unconditional love’ – Cloke *et al*. 2010), but their competence and quality of judgement is likely to vary. Clearly, also, both tensions and synergies exist between them and the big players. The tensions relate to big player dominance within the homelessness industry, which tends to exclude the small players from decision-making and from the general process of service co-ordination, and also to competing interpretations of professionalism. The synergies arise from similar approaches and aspirations towards helping homeless people, and shared recognition of the need for a division of labour between big and small players that reflects the diversity of their clients. However, the tensions between receptivity and non-receptivity to otherness, and between ‘professionalism’ and ‘voluntarism’, exist within every homelessness organisation, whether big or small, and even within the practice of every individual working in the homelessness industry. It seems likely that every individual and organisation is receptive to some ‘others’, but not other ‘others’.

## Conclusion

This paper has shown that homelessness organisations in Stoke formed a community of practice (the Priority Needs Group), within which a consortium of larger organisations acted as the main instrument for co-ordinating inter-agency working. These larger organisations became more concerned with cost-effectiveness due to their funding and contractual obligations, resulting in a more standardised approach focused on clients who could be helped more easily and at less cost. Working alongside them were smaller organisations who provided a more specialist, flexible and personalised alternative, geared to supporting people with multiple and complex needs, who were more difficult and expensive to help and where support was more likely to fail. It seemed that some inter-organisational relationships worked well and the resulting forms of collaboration were held up by respondents as exemplars of good practice (see Cornes *et al*. 2013).

Other cities in the United Kingdom and elsewhere could learn from the common purpose among the homelessness agencies, the degree and quality of co-ordination achieved by the ‘big players’, the general acceptance of a keyworker model, the priority given to multiple and complex needs, and the self-organising of a progressively deepening community of practice. All of this appears to satisfy the aspirations expressed in both the policy and academic literatures on this topic.

Recent cuts in government funding, however, have made both statutory and voluntary organisations increasingly anxious about their financial situation. At the final research advisory group meeting in 2012, fears were voiced that a lack of resources could frustrate joined-up working (Oldman 1997) and threaten the very existence of their organisations. Since then, though, Brighter Futures has won funding from the Big Lottery Fund under the latter's Fulfilling Lives Programme, which it is using, in partnership with other homelessness organisations (both big and small) and service users (‘expert citizens’), to extend and deepen the community of practice in Stoke, for example, through shared databases, assessment, planning and client records across the board, and by developing peer mentoring and evaluation.

We conclude that the revanchist thesis largely explains contemporary homelessness on all scales (local, national and global), but does not explain the ‘ambivalence’ (DeVerteuil 2006) of homelessness organisations, which is corroborated by our case study and is arguably characteristic of life in a capitalist society. Revanchism is a sub-project of neoliberalism, which lacks receptivity to the other (as in ‘there is no alternative’, meaning there is no ‘other’ to a neoliberal project). This receptivity is necessary, though not sufficient, to emancipate homeless people. Effective response to what is received from the other requires willingness and competence to act appropriately, including advocacy on behalf of the other, and continuing dialogue with the other, involving mutual learning and the building of relationship. Through such attitude, skill, action and relationship, homelessness services can become less the servants of capitalism and more the enablers of liberation. Our case study has shown that some homelessness organisations understand the harms wrought by neoliberalism and have found ways to address these harms. At the same time, however, they find themselves increasingly having to play the neoliberal game to survive.
